# Participatory Design Process of a Supportive Digital Platform to Enhance Local Urban Food Systems

**DOI:** 10.3390/foods15152700

**Published:** 2026-07-31

**Authors:** Angela C. B. Trude, Anne-Laure White, Zoya N. Rehman, Ran Mei, William Thomson, Mario Giampieri, Ciara Sidell

**Affiliations:** 1 Department of Nutrition and Food Studies, Steinhardt School of Culture, Education, and Human Development, New York University, New York, NY 10012, USA; aw5163@nyu.edu (A.-L.W.); zoyanrehman@gmail.com (Z.N.R.); rm6723@nyu.edu (R.M.); 2 Share Shed NYC, New York, NY 10012, USA; wdt6208@gmail.com (W.T.); mariogiampieri@gmail.com (M.G.); farmerc.nyc@gmail.com (C.S.)

**Keywords:** digital tools, healthy food access, participatory design, local food system, food security

## Abstract

Digital tools have emerged as one promising strategy for supporting local food systems and strengthening access to food. However, top-down approaches often encounter resistance or low uptake of digital platforms among underserved users because of limited digital literacy, mistrust, or misalignment with on-the-ground realities. We employed a four-phased participatory design process for AgriShare NYC, a digital platform that documents diverse forms of food production across New York city. Through in-depth interviews with 17 growers and three participatory design workshops (*n* = 21), and the development of design criteria, growers’ priorities, capacities, and concerns shaped the platform’s evolution. Growers’ capacity to monitor data was constrained by labor, time, digital literacy, and organizational structure. Simplicity was a guiding principle for the design of this digital platform for urban growers. Thus, the participatory design shifted the purpose of the digital platform from a production-focused data repository towards a connectivity, mutual aid, and advocacy-oriented platform designed to strengthen grower-to-grower connections, meet tangible needs, and make structural gaps visible.

## 1. Introduction

Food insecurity, i.e., the lack of consistent access to sufficient food for a healthy life, remains a critical public health problem in the United States (U.S.) [1]. This issue is deeply compounded by structural and systemic inequities, including rising housing costs, low wages, and historical racialized segregation, which collectively restrict access to affordable, nutritious food [2,3,4]. Nationally, 13.7% of the U.S., i.e., more than 18 million households, were food insecure in 2024, with substantially higher rates among Black and Hispanic/Latino populations [1]. In New York City (NYC), an estimated 1.4 million residents (16.8% of the population) were food insecure in 2023 [5,6,7]. These disparities translate into poorer diet quality, elevated rates of diet-related chronic disease, and greater difficulty complying with basic public health recommendations, particularly in low-income neighborhoods and communities of color [8,9,10]. Grounded in the theory of food justice, systemic food insecurity is conceptualized not as an issue of inadequate food supply, but rather as historical disinvestment rooted in racism that deprives marginalized communities of sovereign control over their food environments [11].

The COVID-19 pandemic made visible these longstanding inequalities and exacerbated food insecurity [12,13]. These trends underscored how heavily individuals depended on consolidated supply chains and an overstretched emergency food network. Within this context, small-scale food producers and urban agriculture initiatives have been increasingly recognized as key elements of resilient, just urban food systems. Urban farms and community gardens facilitate localized access to fresh produce, support informal nutrition education, and strengthen social cohesion, even if their aggregate nutritional yield remains modest relative to total citywide consumption [14]. Concurrently, an emerging body of critical work emphasizes that urban agriculture projects are structurally precarious, relying on unstable volunteer labor and small grants, insecure land tenure, and exposed to urban development dynamics that can undermine their long-term viability [15,16,17].

Digital technologies have emerged as one promising strategy for supporting local food systems, by increasing visibility and spatial navigability of food environments, coordinating stakeholders, and generating data for planning and advocacy. However, experience with digital health and agricultural technologies suggests that top-down, non-participatory technologies frequently suffer from low adoption rates or community resistance. These failures are driven in part by limited digital literacy, institutional mistrust, or operational misalignment with on-the-ground realities [18,19]. To counter these limitations, scholars have increasingly called for equity-centered approaches, specifically participatory design, which particularly involves end-users in the co-creation of the technologies that affect them, ensuring that the final product aligns with users’ lived experiences and work practices [20]. The participatory design approach centers the needs, capacities, and situated knowledge of those most affected by food insecurity and structural inequities [21,22]. According to the design justice framework [23] participatory design is reframed not only as a method for improving user retention, but as a means to democratize technology production by centering frontline growers as active co-designers. Ultimately, this collaborative process transforms users from passive data subjects into self-determining agents of data sovereignty through enhanced visibility, active engagement, and non-discrimination [24].

To address current gaps in the design and implementation of supportive digital infrastructure for urban food systems, this study investigates how digital tools can be equitably and sustainably co-designed with urban agricultural growers and key stakeholders. Specifically, this paper examines the participatory design process of a digital platform to enable collaborative data generation for planning and advocacy, with the broader goal of contributing to a more resilient, community-based urban food system in NYC.

## 2. Methods and Data

### 2.1. The Setting and Context

**Figure 1:**
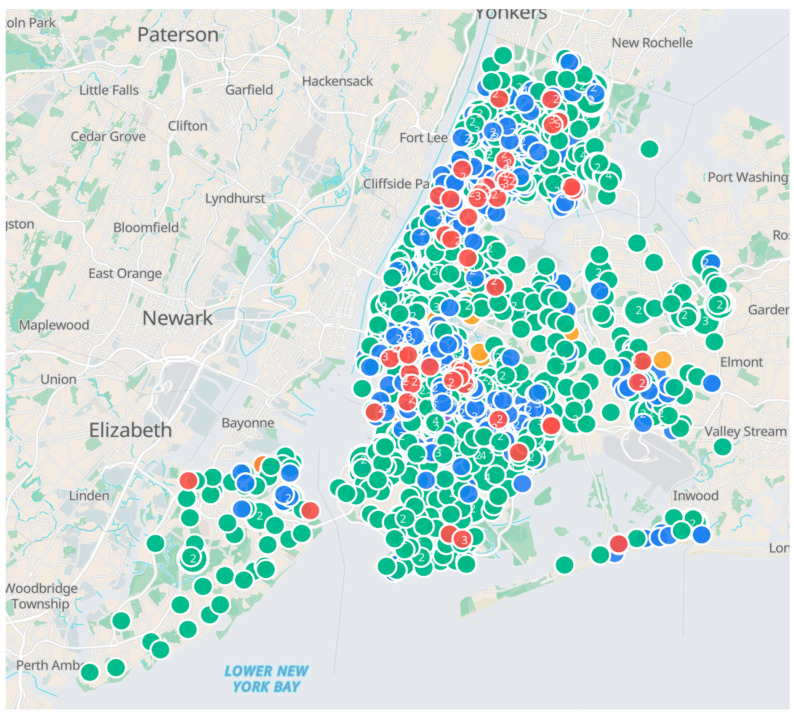
Map of urban agriculture sites in New York City, based on 2026 updates to AgriShare NYC digital data platform.

NYC represents a major urban center for agricultural innovation, encompassing approximately 2580 active urban agriculture sites distributed across its five boroughs (Figure 1). A significant proportion of these sites operate under municipal oversight programs, including NYC Parks GreenThumb, the NYC Housing Authority (NYCHA), or land trust governance structures. The city’s urban agriculture landscape is diverse, comprising neighborhood community gardens, urban farms, school or learning gardens, rooftop farms, controlled environment agriculture (CEA), food forests, and backyard plots [25]. As of 2022, over 550 community gardens, including those part of the GreenThumb and NYCHA coalitions [26], 20 commercial urban farms [26], and more than 800 schools and learning gardens [7], have been reported to make up NYC’s urban agriculture landscape.

Contemporary urban agriculture in NYC has evolved to address social and environmental disparities, demonstrating higher spatial density within historically underserved, low-income neighborhoods [27]. As the nation’s largest urban gardening program with more than 550 community gardens across five boroughs, NYC has long depended on public gardening and urban agriculture as an emergency strategy to support the local food system and strengthen community resilience against social and economic crises, natural disasters, and periods of heightened food insecurity [28,29,30].

### 2.2. Origins of the AgriShare NYC Digital Platform

The AgriShare NYC digital platform originated as Mapping Agricultural Production in NYC (M.A.P. NYC), an interactive crowd-sourced map designed to spatialize food production, distribution networks, agricultural technologies, labor inputs, and community service provisions. Initiated as an interdisciplinary collaboration between the Stern School of Business and the Tandon School of Engineering at New York University (NYU), the project addressed a critical gap in authoritative, spatialized data regarding municipal food production. The urgency to establish an empirical baseline to monitor and quantify the trajectory of urban agricultural growth was further accelerated by the systemic food supply disruptions experienced during the COVID-19 pandemic. The initial deployment of the tool in 2021 generated preliminary spatial data, providing an aggregate overview of NYC’s urban agricultural activity [31].

However, this early iteration relied primarily on top-down data curation mechanisms. Graduate researchers from NYU’s Center for Urban Science and Progress (CUSP) aggregated open-source geospatial datasets to populate the platform architecture. Data parameters were selected through preliminary consultations with agricultural stakeholders, prioritizing macro-level variables suitable for quantifying aggregate crop yields and production capacity. The dataset was composed of open-source data and wiki-style data, in which voluntary users (i.e., growers) contributed information. Consequently, user engagement remained limited; among the approximately 2500 identified farms and gardens on the platform, only 100 entries were directly initiated by agricultural producers.

In light of the urgent need for baseline information to support the development of the urban agriculture sector, the project secured additional funding from the Foundation of Food and Agriculture (FFAR) in 2023 to implement a participatory approach in the redesign of the platform. After input from growers and the project Advisory Board, and subsequent partnership with a volunteer-led organization called Share Shed NYC that focuses on resource sharing, the platform was reestablished as AgriShare NYC. The team consisted of two co-PIs, a project coordinator, a web developer, 3 undergraduate research assistants, and 7 graduate research assistants over the course of three years. The project maintained the Advisory Board structure of the previous iteration, with members from diverse urban agriculture backgrounds, including NYC Parks’ Greenthumb, New York Sunworks, CityHarvest, NYC Food Policy, Randall’s Island Park Alliance, Share Shed NYC, Cornell Cooperative Extension, and GreenMap.

### 2.3. Study Design

To redesign a more equitable and sustainable data-driven digital platform to support growers and other stakeholders in urban agriculture, we adapted and applied a user-centered design [32]. In user-centered design, preferences and needs of anticipated end-users of the product are analyzed early in the process to maximize usability. The future users of the digital platform (e.g., farmers, extension agents, policymakers) are involved in the design process, including identifying the problem, selecting partial solutions, and providing inputs for refining a viable new digital platform. In the redesign and development of the AgriShare NYC app, feedback and suggetsions from high-level users—policymakers and researchers—had already been considered in the digital platform’s 2021 development. Therefore, our redesign process centered around an underrepresented group in urban agriculture—growers, whose use determines the quality of data and value of this wiki-style digital data platform over time. Following a four-step methodology, we moved from a broad problem (Phase 1: Content Exploration) and its context (Phase 2: User Focus) to a specific solution (Phase 3: Design Criteria and Decisions) and evaluation (Phase 4: Pilot Implementation and Evaluation).

### 2.4. Phase 1—Context Exploration:

In the first phase of the design process that took place in 2023, we sought to understand the current landscape of data management practices across NYC’s urban agriculture sites and the relevant constraints encountered by growers. The use of qualitative methods seemed most appropriate for this; therefore, we carried out semi-structured in-depth interviews (IDIs) with a random purposeful sample of urban growers belonging to diverse growing spaces (i.e., community, commercial, educational) across the five boroughs of NYC.

This phase was informed by informal discussions with stakeholders on strategies to include a representative swath of growers from NYC and methods for grower outreach. Recruitment of NYC growers for the IDIs was by random purposeful sampling using a list of 2460 urban farms and gardens collected from open-source and private data during the 2021 iteration of the AgriShare NYC digital platform, which was the most comprehensive dataset available at the time. Using the early version of the AgriShare NYC data, we randomly selected participants from across the five boroughs, with each borough having a number of growing spaces set a priori proportional to their density in the city. We planned to interview about 20–25 farms/gardens or until we reached data saturation: (*n* = 9) gardens in the Bronx, (*n* = 15) in Brooklyn, (*n* = 9) in Manhattan, (*n* = 5) in Staten Island, and (*n* = 7) in Queens. We similarly selected commercial (*n* = 2), community (*n* = 19), NYCHA (*n* = 13), school (*n* = 10), and non-profit/institutional spaces (*n* = 1) based on their relative number of growing sites distributed across the five boroughs. We contacted growers directly via phone/email and followed up for at least three contact attempts, after which we labeled them as not interested.

One-on-one IDIs were conducted both virtually and in-person throughout 2024 depending on growers’ preferences to reduce participant burden and prioritize accessibility. A semi-structured interview guide, designed in 2023, was utilized during IDIs. This interview guide was pilot-tested with a grower who had been involved in the first iteration of the AgriShare NYC digital platform. It was then distributed for feedback among a group of urban agricultural experts that served as an advisory body for the project. The final interview guide included questions on individual garden context and goals, data monitoring and tracking, and experience using the 2021 version of the AgriShare NYC digital platform. Interviewers were trained on a graduate level in qualitative data collection, and completed Debrief Forms after each interview to record the appearance of new or salient themes. Example questions from the interview guide are shown in Table 1.

### 2.5. Phase 2—User Focus:

Informed by the challenges and opportunities identified in the IDIs, we conducted a total of three participatory design workshops in 2024 to understand growers’ experiences with and motivations for using the AgriShare NYC digital platform, and to deepen our understanding of their data-sharing capacities. We included growers falling on distinct points on a spectrum of experience with the digital platform: (i) experienced growers, i.e., growers who either had some experience using or knowledge of the first iteration (i.e., M.A.P. NYC) of the AgriShare NYC digital platform, and (ii) inexperienced growers, i.e., growers with no prior experience or knowledge of the platform. This sampling strategy was determined based on the following factors:

1. Feasibility: growers we could contact;
2. Representativeness: growers belonging to a variety of growing spaces in NYC;
3. Diversity of experience: experienced growers who could offer targeted input, and inexperienced growers who could provide general, context-independent input.

Convenience sampling for the participatory design workshops [33] relied on institutional partners and contacts from events, and listserv signups to contact and invite participants. Some growers were identified and contacted via urban agriculture organizations like Greenthumb. We recruited others by inviting growers to sign up at avenues such as QR codes at conferences hosted by NYCHA, and The Urban Farmer-to-Farmer Summit (TUFFS) hosted by Cornell University, or directly via email through listservs.

Our first two workshops focused on identifying growers’ priorities by encouraging them to envision their “ideal” version of the platform. We first created spaces for brainstorming and ranking priorities of interest. Then we honed in on the design and current structure of the previous iteration of the AgriShare NYC digital platform. Our third workshop focused on resolving discrepancies between defined priorities, grower data-sharing interests, and capacity or challenges to finalize the user focus and inform building of the platform.

All three workshops were conducted in person based on growers’ preferences and to foster discussion among participants. Workshop guides developed in 2024 were used to direct these discussions. These guides moved from general to specific input on the priorities and design of the digital platform and the types of information collected in this digital platform. Examples of workshop questions can be seen in Table 2. We employed an iterative approach, wherein we incorporated lingering questions from previous IDIs (Phase 1) or earlier workshops with participants, but also used these questions and information to move forward in the design process.

### 2.6. Phase 3—Design Criteria and Decisions:

Using findings from the interviews and workshops, we defined several core requirements that needed to be addressed by the design of a data-driven digital platform in collaboration with a professional web developer. Phase 2 and 3 occurred simultaneously and iteratively, with design decisions and prototypes tested in subsequent workshops where we observed and recorded growers’ reactions, behaviors, and misuses when interacting with the mockups. We jointly discussed growers’ perceptions of the various aspects of the digital platform and made decisions on the eventual design.

### 2.7. Phase 4—Pilot Implementation and Evaluation:

We created a beta version of the AgriShare NYC app (v1.3.0; Research Project Studio LLC; available at [https://share.google/9J5uBBDwp5i8KvTtnd] (accessed on 22 July 2026) and tested it with various urban agriculture stakeholders in NYC present at two events: (1) a University-led Summit on Housing and Food Access issues attended by scholars, community farmers, and advocates; (2) GrowTogether Conference organized by the NYC Parks and Recreation Department. During the two events, at least three members of the AgriShare NYC team were present to go over the app with future potential users. All attendees received an initial training on the use of the app with an instructional video and a how-to guide on the navigation of the tool. In all the events, researchers presented the new digital app and encouraged attendees to register in the app and browse through it during the demonstration session. After the pilot, attendees were prompted to answer a brief Qualtrics survey by scanning a QR code with their phones to provide feedback on usability of the app. Attendees were not required to answer the survey, but were highly encouraged at the end of the demo sessions. Evaluation of the app was assessed with the following questions: 1—Did you use the AgriShare NYC app during the demo? (yes, no); 2—How easy was it to use the app? (5-point Likert Scale); 3—How would you improve the app? (open-ended); 4—What would help you to use the app (if answered “No” to the first question)? (open-ended). Following the events, the web developer offered two Office Hours via Zoom to address issues, solicit feedback from potential users and clarify questions. All farmers who participated in either Phase 1 or Phase 2 were sent an email and a calendar invite to join the Office Hours during regular work hours.

### 2.8. Qualitative Data Analysis for Phase 1 and Phase 2

All IDIs and workshops were audio-recorded with participants’ consent and transcribed verbatim. Initial transcription was completed by an online transcription service (rev.com) followed by a manual validation process by a researcher.

Qualitative data analysis of the IDIs took place via coding using principles of thematic analysis [34]. Three researchers developed the codebook iteratively based on line-by-line coding of four in-depth interview transcripts. First, we selected the transcript that provided the thickest and greatest breadth of information based on our Debrief Form and independently used open and axial coding to generate a preliminary codebook. Next, the analytical team met to discuss the codes and generated a unified codebook that detailed when to use and not to use each code. Next, two coders independently applied the preliminary codebook to a second transcript to refine existing codes and identify emerging ones. Then, the analytical team met again to debrief the second transcript. Coding disputes were resolved by a third researcher who would determine which of the codes was most appropriate for the segment or if a new code needed to be developed. Using the refined codebook, the same pair of researchers went back and independently re-coded the two initial transcripts, double-coded another two additional transcripts, with further meetings to dispute codes in between. The team maintained a shared decision trail documenting why codes and themes were merged, renamed, or discarded. After the fourth transcript, the analytical team perceived that the two independent coders agreed substantially on their coding process and moved on to independently coding the remaining transcripts, consistent with collaborative thematic analysis methods [35]. Example codes from the finalized codebook included: Infrastructure, Data, Capacity, Digital Platform, Challenges. Qualitative analysis took place on MAXQDA (version 2022, VERBI Software, Berlin, Germany) [36].

For the workshops, data were analyzed collaboratively with participants during each workshop. Workshop facilitators guided participants in collectively reviewing, refining, and prioritizing key findings, enabling real-time member checking and consensus building. These participant-generated summaries therefore served as the primary data source for Phase 2 and were used to validate, elaborate, and prioritize themes identified during Phase 1. In parallel, we also used the codebook generated in Phase 1 to code the workshop transcripts. However, we noticed the participant-generated summaries provided richer analytical information than generating a separate set of themes. Because the workshops were designed as participatory co-design sessions rather than as additional interview data collection, we opted to prioritize the synthesized data generated during the workshops by the participants and thus analyzed qualitative data directly with the workshop participants.

## 3. Results

### 3.1. Phase 1—Content Exploration

We conducted IDIs with 17 growers from community gardens (*n* = 14), commercial (*n* = 2), and educational spaces (*n* = 1). Interviews took an average of 33.5 min (SD ± 9.5 m) (range: 11 min–47 min) and were conducted in person, with the research team visiting growers on-site (*n* = 14); some were conducted virtually via Zoom (*n* = 2) or over the phone (*n* = 1) in English. For many growers, data management practices were intertwined with material resources, capacity and community support, and the distinct culture and mission of their space.

#### 3.1.1. Data Monitoring Practices and Attitudes

We observed diverse data practices across growing spaces, with unique approaches providing a framework for examining specific kinds of information that growers sought to capture in terms of formal, informal, or none.

Growers engaged in formal data monitoring (*n* = 7), i.e., any data collection or observation that involved a defined set of procedures applied across the farm/garden, with responsible parties and designated infrastructure, employed a range of methods: three used either spreadsheets or written reports; one depended upon specialized software; and three did not specify tools. Precision varied, with some gardens tracking yield by weighing harvest while others estimated or kept informal tallies of crops either grown or distributed. One garden had only recently introduced a weighing system, but the practice was not yet fully established. The kinds of data collected varied according to each garden’s focus and resources (people, time, finances/infrastructure). Five growers tracked produce-related information, though the precision and consistency of these efforts differed. Beyond produce, two gardens monitored honey production and beekeeping activity, while two others maintained composting records. Several growers (*n* = 6) emphasized the social dimensions of their gardens: three tracked volunteer or membership participation, two monitored youth or school group involvement, and one documented partnership with community organizations. In addition, one grower tracked consumer data, aided by the transaction tool Square, which helped them record market attendance and basic customer information.

Those who relied on informal approaches (*n* = 6) tended to monitor data in less structured ways, taking photographs to document harvests or using observation-based summaries to track progress. Among some of these adapted approaches were spaces that had either multiple garden members or organizations engaged in different practices. One such space had an affiliated organization that assumed responsibility for periodic monitoring, leaving the garden with incomplete data in the interim where only the host organization was responsible. These differences in data collection methods, strategies, and accountability represent distinct goals, resources, and attitudes towards data collection. Interviewees repeatedly highlighted the importance of structural factors, such as staff and time, and demographic factors such as age of farm/garden members and degree of community engagement, in determining how or whether to collect data.

A total of four growers interviewed described not having any data monitoring practices, with a commercial grower describing fear of competition as one possible reason for not engaging in such practices: “*The problem with that is a lot of people don’t want to share that stuff because it’s competitive and people have way too much ego about that kind of stuff a lot of the time*.”

#### 3.1.2. Data-Sharing Attitudes

Growers primarily had positive feelings about sharing data, though a fair number were ambivalent, with only one on the mistrustful end and the majority simply less actively engaged with data sharing. Growers also expressed that once they knew what the benefits of sharing information would be, they would be much more open to sharing: “*We’d be interested in sharing it…It depends on the amount of data that we would have to share. And that’s why I was mainly asking if it was public or private because I don’t mind sharing that with growers and stuff. It’s just more like if someone just went to the website and found this stuff and posted a news article about it, I don’t know if I would feel comfortable with that* [data sharing].”

#### 3.1.3. Barriers to Data Monitoring and Sharing

Growers described multiple obstacles to monitoring planting, garden output, food scraps, and consumer or visitor data, most notably the lack of personnel who could be accountable for such recordkeeping. Many gardens operated on a plot-based structure, where each member managed their own bed, making it difficult to track outputs collectively. As one grower explained, “*it’s not very easy to do a whole general garden because everybody is doing their own thing*.” Garden leadership was often not positioned to take this on, as they needed to prioritize on-the-ground work, such as weeding and supporting members, as opposed to data management: “*All of that logistical stuff… takes up my time… it is low on the totem pole for me*.” Beyond limited time and labor, several participants cited low community buy-in, technological barriers for older members, insufficient tools, and a general preference for storytelling over formal data as obstacles to tracking. Among barriers to data monitoring and sharing, the majority of growers identified broader structural needs such as financial support, labor, and connectivity with other growers: “*Most of the time, we want people to work and help us do things because it’s a big place and you always need hands*.”

#### 3.1.4. Urban Growers’ Feedback on the First Iteration of the AgriShare NYC Digital Platform

When presented with an early version of the AgriShare NYC platform on a web browser, growers expressed a mix of enthusiasm and uncertainty about its design, usability, and data priorities (Table 3).

Participants wanted to see information that directly supported their practices—such as funding strategies, distribution sites, specializations on other farms that they could connect to or learn from, resource sharing, student or skill-building programs, and composting systems—and to know how they might add, correct, or update data over time. Participants also wanted to see data about other aspects of the food supply chain in NYC, namely food access points such as pantries and free neighborhood fridges. Collectively, this feedback highlights growers’ vision of digital data platforms not as static repositories but as living, participatory systems for connection, learning, and accountability within the city’s food-growing community.

Many growers who found the digital platform difficult to navigate, for example, were also interested in certain filters. Five growers specifically suggested clearer category definitions and more intuitive ways to toggle filters on and off. Some participants (*n* = 4) noted that the name, “MAP NYC,” did not clearly convey its purpose or focus. Using a computer was not easy for all gardeners, so the idea of a mobile application recurred: “*An app would be good*.”

#### 3.1.5. Facilitating Factor for Using Digital Data Platforms

Despite challenges with tracking and sharing data, most growers expressed excitement about collaboration and resource sharing with peers. The possibility of knowing what other gardens were producing, where they were distributing, and what kinds of skills could be shared from grower-to-grower was a major motivating factor in using a digital data platform.

Participants described interest in exchanging technical knowledge, physical materials, and management practices—ranging from composting and soil health to funding strategies, CSA (Community Supported Agriculture) participation, and volunteer coordination. Growers often had direct skills they hoped to share: “*When you get a fungus on all of your squash, why? How do you get rid of it?*”

Others highlighted opportunities for sharing tangible materials such as seeds, soil, and tools. A school gardener in Brooklyn suggested coordinating production across gardens to pool resources and serve their communities collectively: *“I’m interested in learning how each of us could offer a different resource to the public, whereas it’s nice for each garden to be able to have a chicken coop and a greenhouse, but not everybody can. Some gardens are not large enough, and everybody doesn’t want to be a beekeeper, so it’d be nice if, within the district, we’d have five who agreed to be beekeepers and five who agreed to have the chicken coop and so forth.”* While many were ambivalent about formal data collection, at least twelve growers indicated they would value access to information about other growing spaces, suggesting that data sharing remains most compelling when tied to tangible collaboration and mutual benefit.

Indeed, some growers emphasized the importance of political or financial infrastructure, while some highlighted the value of mutual dependency across the urban agricultural community. These two perspectives were not mutually exclusive. Many growers who wanted more structural support were also interested in grower-to-grower sharing. Data sharing was seen as valuable insofar as it could be a tool to expand capacity: “*I feel like information sharing is really important in these kinds of spaces, like I’m saying with skill sharing, along with that, because we can’t all do everything and we can’t do it alone*.”

### 3.2. Phase 2—User Focus

We convened three workshops with an average of seven participants in each (range 6–8) spanning community growers, educational growers and institutions. Collectively, our workshops included a total of 21 participants. The first workshop included eight participants with some previous user experience of the first iteration of the digital platform, including four community gardeners, two non-profit gardeners, one non-grower from an urban agricultural support organization, and one grower at an educational institution. The second workshop, with six participants, was for inexperienced users and included one community gardener, one gardener at NYCHA, two non-profit growers, and two commercial growers. The third workshop brought together seven inexperienced and experienced users from four community gardens and three institutional/non-profit growers.

**Figure 2:**
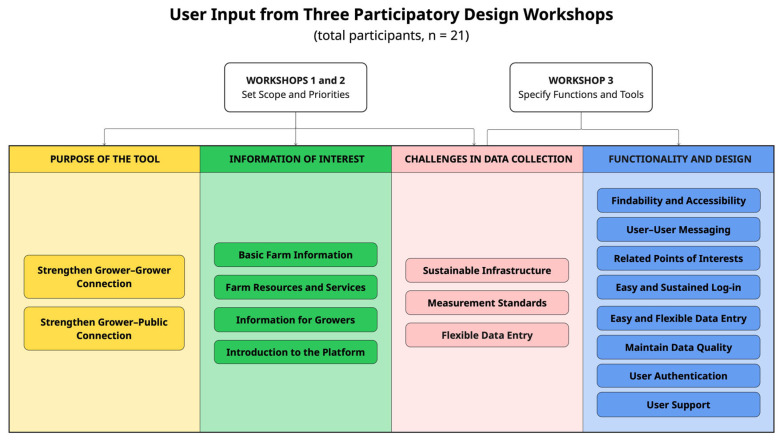
Grower input across three participatory design workshops.

Workshops occurred over an average of 2 h and 12 min, with the earliest workshop at nearly 3 h and the next two at an average of 1 h and 48 min. Findings from the workshop and key takeaways are summarized in Figure 2.

#### 3.2.1. Purpose of a Digital Data Platform

Participants across all three workshops voiced many common priorities for digital data platforms that support urban food systems. A main data priority was to facilitate access to resources and infrastructure for farms and gardens. Growers suggested strategies varying from grower-to-grower connections to demonstrations of impact to external parties capable of shaping policy, furnishing funds, and otherwise supporting the spaces. All participants expressed hope that their participation in such digital data platforms would result in tangible gains, either through connections with other growers or through an external demonstration of their impact and resource gaps to policymakers: *“I think to be able to do partnerships with various partner gardens: events together, even grant applications together would be great. So, I think having a way to list out what type of partnerships that you’ll be open to would be really, really interesting as a data point*”. Some participants also indicated a desire to have continued meaningful participation through sustained engagement with the digital data platform, such as AgriShare NYC, and suggested outreach strategies to facilitate this.

#### 3.2.2. Information of Interest for Inclusion in the Digital Data Platform

Beyond broad thematic priorities, participants suggested specific questions and concrete information that they would find valuable. Nearly all participants across the three workshops emphasized the importance of including basic information on each farm or garden, including name, address, type of organization (i.e., community, commercial, school), institutional affiliations, hours of operation, accessibility, size, and mission, as well as contact information.

Participants were interested in a bidirectional resource-sharing approach, in which a farm or neighborhood group could share resources they had in excess, as well as advertise what they might need from others. Some possible resources for exchange included composting, seedlings, and soil. Useful information that might facilitate resource-sharing outside of the growing community included the locations of community fridges/food pantries. This emphasis on mutual aid was also met with an interest in more structural needs, such as increased funding or access to water.

In terms of technical garden information, participants thought specifics on growing environments, growing practices and methods, yield, and distribution channels were valuable. Moreover, some participants demonstrated interest in aggregated data such as percentage tree cover, percentage rainwater catchment, and percentage pollinator habitat.

#### 3.2.3. Input on Functionality and Design (UI (User Interface)/UX (User Experience))

Participants’ testing of the digital platform offered users the opportunity to put forward their perspectives on it and provide user-driven input to improve it. Practical feedback on each stage of the user experience and interface emerged through discussions, covering the sign-up process and data entry, and touching on transparency of data collection methods and sources. The primary need for UI/UX was simplicity.

#### 3.2.4. Sharing Garden Data: Interest, Feasibility, and Challenges

Discussions among participants highlighted the incongruity between their priorities and capacity to implement them, underscoring the notion that desire does not equate with feasibility. Although participants agreed that crop production and growing methods were foundational to a digital platform that supports urban food systems, a number of challenges to collecting such data were discussed. Time constraints, different levels of digital capacity across generations, and a lack of appropriate infrastructure for collecting and sharing data were among the core obstacles.

In response to these challenges, we tried to produce sustainable digital infrastructure that created a measurement standard while also allowing for special cases. In practice, this looked like allowing space for growers to select multiple responses to a question, as well as leaving an option for growers to select “other” and expand on their response in a text box. When it came to yield, we made sure that this was not an essential question and instead made it essential to fill out information on what kinds of crops were produced. Individuality and simplicity were core characteristics of the design, though accomplishing them sometimes required compromises and flexible solutions.

### 3.3. Phase 3—Design Criteria and Decisions

Based on findings from interviews and workshops with growers, the AgriShare NYC team developed new design criteria along with the team’s web developer. These changed both the functional components of the platform (navigation, layout) and the content (data type, filters). In all design decisions, simplicity appeared as a guiding principle, creating both opportunities and constraints.

In participatory design workshops, the distinct needs of diverse kinds of growing spaces led to brainstorming dynamic and adaptable solutions in the design. Just as accommodating individual garden needs did not always align with the overall priority of simplicity, the outcome of participatory design did not always align with the parameters of development of the digital platform. For example, there was interest in including data on food bank locations. This data was ultimately not feasible to include, as current food bank maps of NYC are highly dynamic and require constant maintenance that the AgriShare NYC team did not have capacity for.

Ultimately, the design input from workshops and interviews revealed a desire for an all-encompassing digital platform. Growers have many disparate resources at their disposal, but were eager for a digital platform that brought together multiple functionalities and auxiliary services. For usability, however, it was necessary to limit the scope. For future technologists, planners, designers, and public health practitioners working in the world of urban agriculture and related digital infrastructure, this desire for consolidation of digital resources should be considered.

One of the most significant changes in the digital data platform was the creation of an accompanying app. After hearing from many growers that computers were either inaccessible or difficult to use in the field, we decided to create a mobile application. This format also encouraged further simplicity, as the data-entry procedure of the wiki-style format had to be made more concise for the smaller screen. In light of the diverse demographics of growers in NYC, including people of many different ages, language backgrounds, and digital fluency, data entry was kept simple. Options for offering on-the-spot Spanish translations were also explored for post-launch.

**Figure 3:**
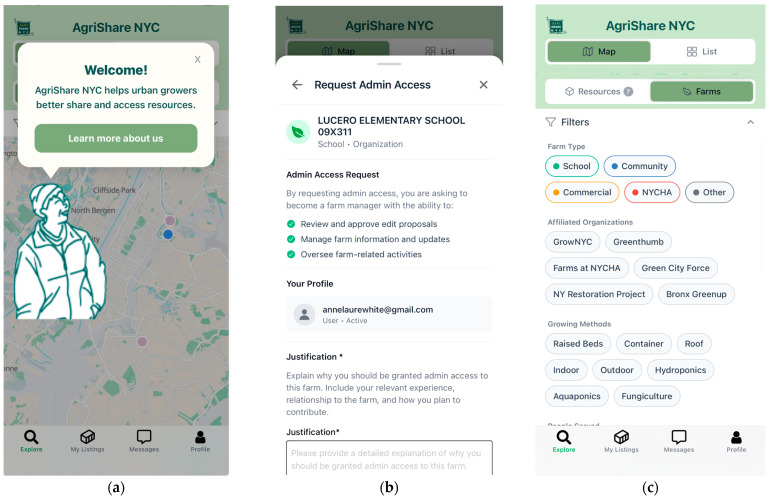
Simplicity was foregrounded in all aspects of design and functionality including from left to right: (**a**) the landing page, which succinctly introduces users to the platform; (**b**) a clearer pathway for questing administrative access to edit farm/garden data, with the option to enter data before receiving approval to streamline the process; and (**c**) a smaller number of filters to choose from, with a simplified filter selection process. *: Required data field.

**Figure 4:**
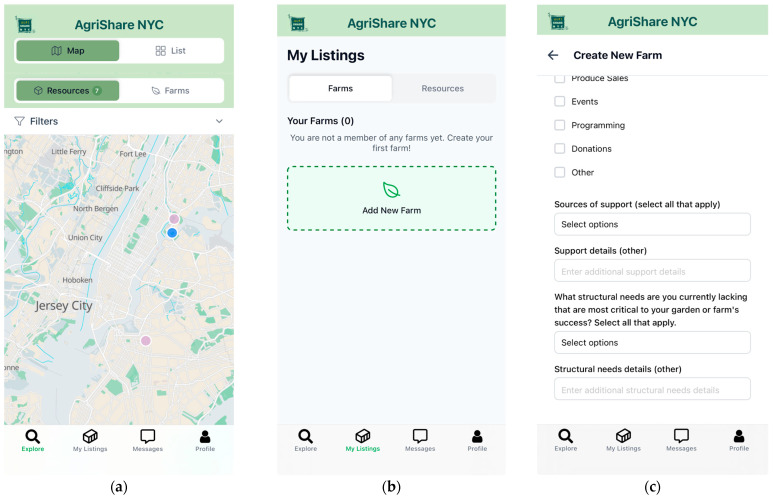
Growers identify that facilitated access to tangible resources is an important priority and incentive for using the digital data platform. In practice, this meant (**a**) integrating resource sharing seamlessly into the map; (**b**) allowing growers to post resources, as well as manage data about their farms/gardens; and (**c**) including questions about structural needs and resource gaps to highlight priorities for policymakers, nonprofits, and private sector urban agricultural investment.

**Figure 5:**
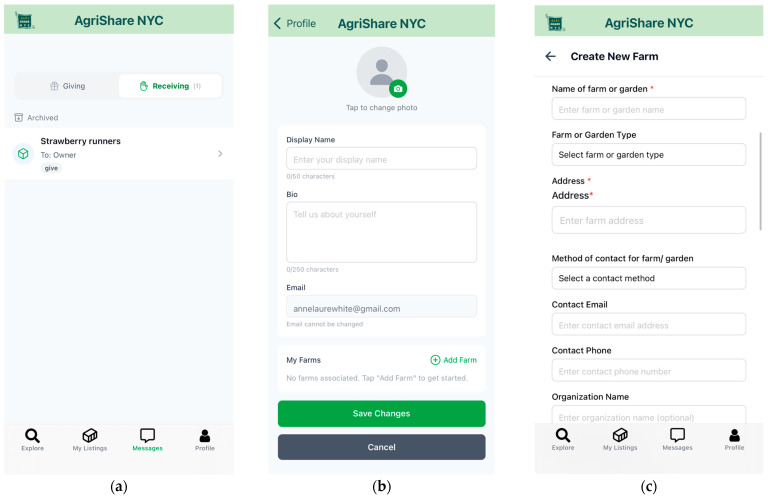
Growers want to be able to communicate with one another more easily. This priority is complicated, however, by privacy concerns. We therefore developed (**a**) a messaging platform where growers could securely exchange messages and information about resources they are giving or receiving; (**b**) a space for growers to voluntarily share individual information; and (**c**) an option to add a farm/garden contact method only if desired by the point person for that space. *: Required data field.

**Figure 6:**
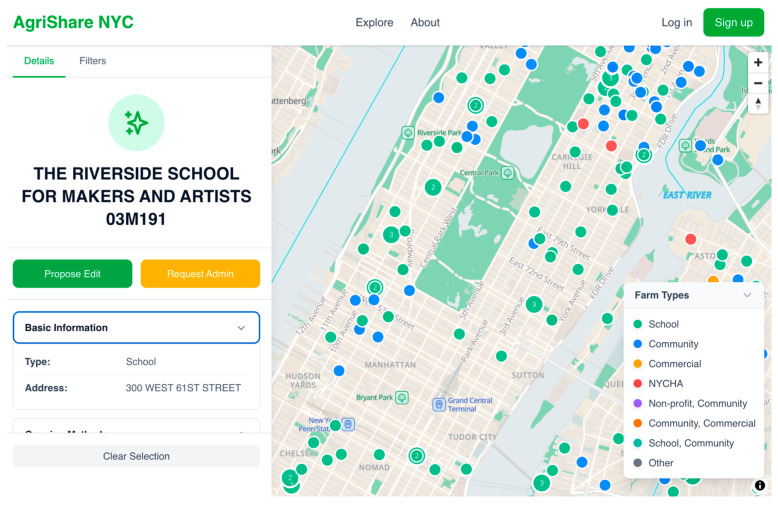
Simplicity also dominates design decisions for the web version of the new AgriShare NYC digital data platform. Options to propose edits, request admin access, and view basic information and filters are foregrounded. The legend is clarified, and the color code is reduced to farm types so as to minimize confusion.

Growers emphasized resource sharing as a key priority for the local urban agriculture in NYC, as well as noting that they had limited capacity for engagement with digital platforms. We therefore opted to minimize redundancies, partnering with an existing group, Share Shed NYC. This volunteer-led organization had similarly engaged growers in interviews and workshops to determine how best to meet interests in resource sharing. Our design process was highly collaborative, as we opted to integrate our two platforms into one, and this was when the new name for the digital platform emerged as AgriShare NYC. The transformation of grower priorities into specific design criteria is elucidated in the development of the digital data platform (Figure 3, Figure 4, Figure 5 and Figure 6).

### 3.4. Phase 4—Pilot Implementation and Evaluation

During the pilot implementation, a total of 2225 urban growing spaces were included in the map either through open data source or manually by a farmer. At the end of the pilot phase 103 individual users registered with an account at the AgriShare NYC app and nine resources were posted by users. Examples of resources to give out were “grafted apple tree”, “African eggplant seedlings”, and “woodchips” and a resource to lend was “shovel for moving mulch”.

Although the platform gained many new users during the pilot testing, not many users provided feedback using the QR code for the Qualtrics survey after the two demo events. We received a total of six responses to the Qualtrics survey, four from the first event, and two from the second event. Of those, five reported using the AgriShare NYC app during the event, and one did not use the app but “wanted to learn more about it before trying out”. Among those who tried the app during the event and submitted their written feedback, two felt the app was “somewhat difficult” due to not being available for Android phones, and another two rated as “extremely easy”. One respondent did not provide feedback on usability.

During office hours, a total of eight farmers joined the virtual meeting with the web developer, who helped troubleshoot issues navigating the app, from the user side and from the app itself. Issues reported were related to visibility where the mobile keyboard blocked critical parts of the screen, preventing users from seeing what they were entering. Another issue reported was related to the legend vanishing as soon as users entered the filter view. Lastly, some users were confused by the “Explore” button and suggested renaming it to “Home” for clearer navigation.

## 4. Discussion

This study advances food system scholarship by exploring how participatory design can build digital infrastructure with urban growers—key actors in improving food security and food access for underserved communities. By connecting farmers to one another, to resources, and generating data for planning and advocacy, this digital tool has the capacity to be a supportive infrastructure that serves the needs of urban growers, policymakers, and researchers to improve local urban food systems. The causal pathway linking this digital platform to food security and food access outcomes is indirect and mediated by the empowerment of local growers. By facilitating peer-to-peer resource sharing, the platform lowers operational barriers for urban farms, thereby increasing their local production capacity and stability. Generating centralized, grower-led data further equips advocates and policymakers with the empirical evidence needed for spatial planning and targeted policy. Although the platform itself does not directly impact food access, it strengthens the organizational capacity, resource flow, and advocacy power of the stakeholders who directly shape the local urban food system. Through this co-designed supportive infrastructure, the digital tool aligns with the immediate priorities and capacities of urban growers for a more equitable and just food system and integrates the foundational principles of visibility, active engagement, and non-discrimination to achieve data sovereignty [24].

Across interviews and workshops, three central findings emerged. First, while most growers were willing to share information about their farms’ resources and services with fellow growers and the public, they were concerned about their capacity to systematically collect, update, and maintain these data with limited labor, time, digital literacy, and organizational infrastructure. Second, engaging with growers in the early stages of the design process shifted the platform’s purpose from mapping sites of urban food production to fostering grower-to-grower connections, resource sharing, community advocacy, and mutual aid while making growers’ needs visible for policymakers and resource-holders. Third, urban growers expressed a strong preference for a simple and accessible platform design that they could easily navigate and interact with. Together, these findings offer a methodological framework for community-based participatory design of digital tools that support accessible and equitable provisioning of healthy foods to ultimately prevent chronic diseases and food insecurity for underserved populations.

A growing body of scholarship recognizes that producing equitable digital health and food system platforms requires an equitable design process [37,38]. Reviews of digital health design highlight that marginalized communities are frequently positioned as end-users rather than co-creators, which often results in tools that reproduce power imbalances and fail to address structural barriers in health and nutrition education, food access and food security, and digital literacy [18,39]. In agricultural and food system contexts, participatory approaches to design digital platforms, traceability systems, and data dashboards with smallholder farmers and community organizations reveal tensions around data ownership, labor burdens, and alignment with local priorities [38]. Our participatory design workshops likewise identified tensions between local priorities and top-down measures. Among growers, many key priorities were shared, but tensions arose between the types of information participants valued and the types of information that they had the capacity to share. Existing studies on mobile apps and web-based platforms related to the food environment similarly suggest that information provision alone does not guarantee uptake or behavioral change, particularly among underserved users facing time, resource, and literacy constraints [40]. Building capacity necessitates building common goals, addressing structural barriers, and educating around digital data platform usage.

Studies of urban food system mapping and inventory digital platforms show that spatialized, publicly accessible data can support planning, advocacy, and residents’ ability to locate healthy food options, but also reveal gaps in coverage and uneven data quality, especially in low-income neighborhoods [41,42]. While such data could be helpful for public health and nutrition policymaking and community organizing, previous studies have shown that urban growers were disinclined to participate when the overall goals of data collection and sharing were unclear [43] and did not align with their everyday tasks and needs [44]. Solutions for encouraging digital data platform use included presenting clear benefits of data-sharing to farmers, strengthening and increasing farm connectivity, and creating a supportive and inclusive data-sharing and data collection infrastructure, which will encourage farmers to actively engage with these digital tools that are critical to building a healthy and equitable food system [43].

Research on digital platforms cataloguing urban agriculture highlights their potential to quantify food production, document the diversity of growing spaces, and inform urban planning and public investment decisions, while noting that informal and community-led sites are often undercounted or poorly described [45,46]. With such potential benefits in mind, findings from our IDIs helped reframe the goals of the digital data platform for clarity and alignment with grower interests. Growers emphasized access to resources as a top priority for a digital platform to promote healthy food access. Such resources included funding, consistent labor, infrastructure, and political recognition. Although sharing food production data was not perceived as the top priority for urban growers, it was seen as potentially useful if it facilitated tangible gains such as support for grant applications and policy advocacy. These dual goals—facilitating peer-to-peer exchange resources while also bolstering funding streams or institutional support—reflect the ways in which urban agriculture’s impacts can be both highly local and contingent upon a city- and state-level infrastructure to address equity in healthy food access and food security.

Findings from the interviews and workshops indicated how central data about food production, visibility, and governance are to debates about urban agriculture and local food systems. Empirical studies of urban agriculture mapping and monitoring show that what gets counted—crop yields, number and size of gardens, organizational forms—shapes how policymakers and funders perceive the sector’s contributions and allocate resources [45,47]. Critical analyses warn that narrow, production-focused metrics can obscure the social, educational, and political work of urban agriculture and may privilege projects that conform to dominant performance criteria [16,48,49]. Our study strengthens these arguments by showing urban growers in NYC placed emphasis on alternative metrics such as community resilience, culturally specific food access, neighborhood beautification, and healthy food education, which are often overlooked by top-down measures to support urban agriculture.

Participants emphasized the need to balance comprehensive data collection with a simple and accessible interface, highlighting a common tension in designing digital tools for community-based health education and outreach between expanding functionality and maintaining usability. The significance of the latter is built into Helbing and colleagues’ definition of “Democracy by Design,” wherein they emphasize that the right to access and share information includes being able to do so with “reasonable effort” [50]. In a recent study applying participatory design workshops and interviews with older adults, a simple interface was the most important characteristic of a digital tool [51]. The importance of simplicity, positive feedback (i.e., accomplishing goals easily and with minimal error messages), proactivity (notifications, for example), and integration (including multiple functionalities into one system) [51] were also highlighted as a design that promoted equity, corroborating findings from our study. Similarly, AgriShare NYC applied all of these principles to design, including simplifying the interface, adding a notification system for updating data, and integrating the resource-sharing component through collaboration with Share Shed NYC.

These design changes reflected a broader shift in the purpose of the digital platform. Rather than serving primarily as a repository for urban food production data, the redesigned platform functions as a bridge that connects growers with fellow growers, as well as between growers and policymakers who aim to improve food access and food security for underserved communities. Similar dynamics have been observed in the application of participatory design to other digital agriculture initiatives, where digital platforms are expected to simultaneously support farmer collaboration and generate data legible to external decision-makers [52]. By including questions about resource gaps alongside food production practices, the digital platform attempts to surface structural needs without imposing excessive reporting burdens, while producing meaningful information for growers and policymakers to improve the urban food system.

Questions of data, visibility, and governance are central to debates about urban agriculture and local food systems. Research on governance and knowledge integration in urban farming initiatives finds that participatory monitoring and co-produced indicators can make structural constraints and resource needs more visible, while creating pathways for growers and community organizations to influence planning and policy to make an impact on food security [53,54]. The participatory design process used to redevelop and transform M.A.P. NYC into AgriShare NYC served to bring the needs and priorities that growers deemed most important to the forefront of the digital data platform, thereby ensuring that future research and use of the data by policymakers would reflect information that the growers themselves valued. These findings underscore that digital platforms for mapping and monitoring urban agriculture are not neutral; they embed choices about what counts as valuable, whose labor and knowledge are visible, and how responsibilities for food data collection and maintenance are distributed. Participatory approaches to digital platform development can help align policy approaches to food access and equity with the values of urban food producers.

During the one-month pilot phase, more than 100 new users successfully registered for the AgriShare NYC digital platform. We consider this rapid adoption rate a preliminary indicator of improved usability, directly attributable to the simplified registration process as a result of the participatory design. Community engagement was further driven by step-by-step demonstrations at two community events with diverse audiences, which served as both outreach and real-time usability testing sessions where qualitative feedback was gathered. Conversely, the Office Hours with the web developer were underutilized by active and prospective users. While this low attendance may suggest a lack of critical software issues or simply reflect scheduling conflicts, the targeted feedback that was collected during these sessions was documented and immediately resolved. We acknowledge that this pilot phase relied primarily on qualitative adoption indicators rather than validated usability scales (such as the System Usability Scale) or longitudinal retention metrics. Moving forward, the research team will collaborate with Community Board partners to distribute a newly developed “how-to” guide to sustain engagement. Future research will implement formal, quantitative frameworks to measure long-term user retention, data quality improvements, and the platform’s ultimate impact on local food access outcomes.

Long-term usability of a digital platform was another key theme from this participatory design study. Thus, once beta-testing is complete, the AgriShare NYC app will transition into a self-sustaining, low-cost operational model housed entirely within the university’s permanent infrastructure. The sustainability strategy focuses on eliminating dependencies on external paid labor by operationalizing a localized “train-the-trainer” approach embedding the app into the university curricula of food systems and sustainability disciplines. Students will use the platform to guide required field interviews with city growers, inputting production methods and resource needs directly into the database to inform their core course papers.

A primary strength of this study lies in its iterative, multi-phase design, which allowed grower input in the form of one-on-one interviews, workshops, and eventually, testing and implementation of the digital tools with users, to shape both data entry and platform functionality over time. Combining interviews with participatory workshops enabled identification not only of priority data categories but also of capacity constraints and usability concerns.

However, several limitations should be noted. The sample size, while diverse across boroughs and growing types, was limited and may reflect a self-selection bias toward growers already interested in collaboration or digital engagement. This mirrors limitations recurrent across other participatory design efforts [55]. The study is also geographically specific to NYC, where urban farm density and public health and nutrition education and policy landscape may differ from other urban contexts. Therefore, findings from this study should be interpreted with caution and may not be generalizable to other populations or contexts, such as commercial or institutional growers who were less represented in this study. This limitation is inherent in qualitative research methods that aim to provide more contextual and in-depth understanding of the topic under study via non-probability sampling techniques rather than numerical generalization. Nevertheless, our purposive sampling attempted to mirror the prevalence of different types of growing spaces across all the five boroughs aiming for purposive variations to enhance transferability of the findings. Finally, some limitations may be inherent to participatory design itself. While the stated goal of participatory design is to produce a digital platform that reflects the priorities and technical capacities of users, interviews and workshops suggested that such methods functioned more reciprocally. Researchers, by educating users on the data platform and engaging them in conversation, reinforce the importance of the big-picture mission and make the project more familiar and, thus, usable [56].

The findings from this study establish the foundational framework for the first design iteration of the AgriShare NYC platform. Furnished with the insights generated during this research period, the platform can be refined and improved. Future research must integrate the perspectives of a broader cross-section of food system stakeholders, specifically commercial and institutional growers, and policymakers to ensure systemic alignment of the digital platform. Second, prior to scaling, a rigorous quantitative assessment of the platform’s usability utilizing validated psychometric scales is required to triangulate and extend the initial qualitative insights presented in this study. Finally, evaluating the long-term impact of the AgriShare NYC tool on urban food systems will require sustained community engagement strategies to drive platform adoption among growers. Concurrently, the proposed pedagogical model, which leverages student-led integration to bridge theoretical frameworks and practical application within higher-ed agricultural curricula, must be systematically evaluated for educational and operational efficacy.

## 5. Conclusions

In conclusion, this study demonstrates the value of employing a participatory design process to engage underrepresented stakeholders, such as urban growers, within food systems research. By centering these perspectives, the study operationalized a design justice framework to identify critical platform capabilities that ensure equitable digital access, participation, and inclusion. Furthermore, this participatory approach directly shaped the functional architecture of the AgriShare NYC platform, transitioning its core utility from a conventional, production-centric data repository to a dynamic network dedicated to connectivity, mutual aid, and community advocacy. Finally, the pilot phase garnered active engagement from over 100 individual users, indicating high platform accessibility and usability. These findings underscore the potential of decentralized digital tools to enhance resource and information accessibility among growers, ultimately contributing to the resilience and optimization of local urban food systems.

## Figures and Tables

**Table 1:** Snippet of questionnaire developed for one-on-one interviews with farmers and gardeners.

Topics	Questions and Probes
Data monitoring and sharing	1. What information do you currently track? Probe: How do you track this information? 2. What information would you like to track? Probe: What keeps you from tracking this information? 3. What are you interested in learning about other growing spaces?
AgriShare NYC app questions	4. As a user and owner of this digital platform, you would be able to upload and download information. What information would you like to share with other growers or interested parties? Probe: How do you feel about sharing information? Probe: Are there limitations in your ability to share certain kinds of information? 5. What would you change to make this tool more useful to you? Probe: Earlier, we discussed reservations about sharing information. In what ways could this tool be altered to address those concerns?
Strategies for engagement with the digital tool	6. What challenges might you face when uploading information about the garden/farm? Probe: time, internet, knowledge, capacity 7. How would you address this?

Note: We use the term “information” rather than “data,” as feedback from experts and pilot tests reveal that the word “data” connotes top-down approaches and homogenization of experience.

**Table 2:** Example of questions used to guide the three workshops.

Level of Input	Questions from Workshops #1 and #2: Priority Building	Questions from Workshop #3: Digital Platform Building
Broad perspective of urban agriculture in NYC	Based on your previous use of this tool, what would be its main purpose for you?	Create a Venn diagram between information growers think would help their farms/gardens reach their goals better and insights they would like to share with policymakers
Specific to AgriShare NYC tool	2. What is every sort of information we might want in a tool relating to food production in NYC?	2. How would your farm elect a person to be responsible for data entry?
Oriented to design and UI/UX of AgriShare NYC tool	3. In order to make sure that the tool actually reflects our interests, we are going to rank these priorities as high, medium, or low. 4. In what ways do these priority items help accomplish the purpose of the tool as originally shared earlier in the conversation?	3. How frequently would you or someone from your farm do data entry? 4. What would make it more appealing to come back to the site and update/enter information?

**Table 3:** Key themes of user feedback on the first iteration of AgriShare NYC.

Theme	Description	Subtheme	Illustrative Quotations
Design	The User Interface (UI), i.e., the visuals of the app including but not limited to layout, structure, colors, typography, etc.	Positive	“Generally positive. [...] Interesting to see the [farm] types, obviously the key is clear, the basic user interface worked cleanly for me. Loading it on mobile worked pretty fast, [...] Pretty straightforward, my first impression.” [ID 52001, non-commercial]
		Negative	“There’s a lot going on. [...] This is a little confusing.” [ID 52004, non-commercial]
Functionality	The User Experience (UX)—the overall experience of interacting with the app in relation to usability, features, etc.	Existing features	“I could filter this. I could see who is doing CSAs, who’s doing farmers markets, donation. I guess that would be just fine for... Probably for research, if we wanted to see who my competitors maybe are. Or for inspiration. What kind of student programs they offer. Income sources. That’s cool. Renewable energy. Yeah. I guess I would use it for research.” [ID 11003, commercial]
		Proposed features	“You should also include things, like… is the garden safe? Not safe from danger, but is it safe from being taken away and plowed under?” [ID 32005, non-commercial]
Information of interest	Data on farm characteristics that were considered valuable by growers to foster connectivity and grower-to-grower sharing of knowledge, resources, and best practices.	Contact information	“Having also a point of contact kind of option, like maybe a button that’s like, ‘Oh, email this garden,’ or that goes to their social media. Some gardens have really active Instagrams, and some are run by elders, so they only have a phone number. So just the best way to contact the garden I think is helpful.” [ID 5I4003, institutional]
		Affiliations	“I mean, what I would want is a Greenthumb filter, or Brooklyn Botanic sponsors community gardens through some other initiative. That’s my entree to like, oh, we’re a Greenthumb garden, you’re a—whatever it is—Brooklyn something garden.” [ID 52001, non-commercial]
		Miscellaneous (including funding, growing practices, non-food production, composting, etc.)	“I want to know all that. Student programs, we get more students into our garden. Environment. How is this going to affect the environment? Does it do a good job? Does it help or does it not help? And people should know where our garden is, and what we give growing methods.” [ID 42001, non-commercial]

## Data Availability

The data presented in this study are available on request from the corresponding author due to privacy and ethical restrictions, as interview transcripts contain information that could compromise participant anonymity.
